# Full genome characterization and evolutionary analysis of Banna virus isolated from *Culicoides*, *mosquitoes* and *ticks* in Yunnan, China

**DOI:** 10.3389/fcimb.2023.1283580

**Published:** 2023-11-16

**Authors:** Zhenxing Yang, Yuwen He, Yiju Chen, Jinxin Meng, Nan Li, Susheng Li, Jinglin Wang

**Affiliations:** ^1^ Yunnan Tropical and Subtropical Animal Viral Disease Laboratory, Key Laboratory of Transboundary Animal Diseases Prevention and Control (Co-construction by Ministry and Province), Yunnan Animal Science and Veterinary Institute, Kunming, Yunnan, China; ^2^ School of Public Health, Kunming Medical University, Kunming, Yunnan, China

**Keywords:** Banna virus, full genome, phylogenetic analysis, *Culicoides*, *mosquitoes*, *ticks*, China

## Abstract

**Introduction:**

Banna virus (BAV), a potential pathogen that may cause human encephalitis, is the prototype species of genus *Seadornaviru* within the family *Reoviridae*, and has been isolated from a variety of blood-sucking insects and mammals in Asia.

**Methods:**

*Culicoides*, *Mosquitoes*, and *Ticks* were collected overnight in Yunnan, China, during 2016-2023 using light traps. Virus was isolated from these collected blood-sucking insects and grown using *Aedes albopictus* (C6/36) cells. Preliminary identification of the virus was performed by agarose gel electrophoresis (AGE). The full genome sequences of the BAVs were determined by full-length amplification of cDNAs (FLAC) and sequenced using next-generation sequencing.

**Results:**

In this study, 13 strains BAV were isolated from *Culicoides*, *Mosquitoes* and *Ticks*. Their viral genome consisted of 12 segments of double-stranded RNA (dsRNA), and with three distinct distribution patterns. Sequence analysis showed that Seg-5 of four strains (SJ_M46, SJ_M49, JC_M19-13 and JC_C24-13) has 435 bases nucleotide sequence insertions in their ORF compared to other BAVs, resulting in the length of Seg-5 up to 2128 nt. There are 34 bases sequence deletion in Seg-9 of 3 strains (WS_T06, MS_M166 and MS_M140). Comparison of the coding sequences of VP1, VP2, VP5, VP9 and VP12 of the 13 BAV strains, the results show that VP1, VP2 and VP12 are characterised by high levels of sequence conservation, while VP9 is highly variable, under great pressure to adapt and may be correlated with serotype. While also variable, VP5 appears to be under less adaptive pressure than VP9. Additionally, phylogenetic analysis indicates that the 13 BAV strains locate in the same evolutionary cluster as BAVs isolated from various blood-sucking insects, and are clustered according to geographical distribution.

**Conclusion:**

The data obtained herein would be beneficial for the surveillance of evolutionary characteristics of BAV in China and neighboring countries as well as extend the knowledge about its genomic diversity and geographic distribution.

## 1 Introduction

Banna virus (BAV) is the prototype member of the genus *Seadornavirus* in the family *Reoviridae*, and it is carried and transmitted by bloodsucking insects (Attoui et al., 2000a). BAV is a segment double-stranded RNA (dsRNA) virus, and the viral genome is approximately 21 kb in length and consists of 12 segments of dsRNA which encode 7 structural proteins (VP1, VP2, VP3, VP4, VP8, VP9 and VP10) and 5 non-structural proteins (VP5, VP6, VP7, VP11 and VP12) (Attoui et al., 2005; Jaafar et al., 2005b). Both VP4 and VP9, which are encoded by segment 4 (Seg-4) and Seg-9 respectively, are the outer capsid proteins of the virus, and may be involved in virus attachment and penetration of the host cell during the initiation of infection (Jaafar et al., 2005a; Jaafar et al., 2005b). The viral inner core with a relatively smooth surface appearance and is made up of VP1, VP2, VP3, VP8 and VP10. In addition to VP7 and VP12, which perform the functions of protein kinases and RNA-binding proteins, respectively (Jaafar et al., 2005b), the functions of other non-structural proteins remain unknown.

BAV was first isolated from cerebrospinal fluid and sera of human patients with encephalitis in Xishuangbanna, Yunnan province, China in 1987 and has been considered a possible human pathogen associated with viral encephalitis and fever (Xu et al., 1990; Attoui et al., 2005). Since then, BAV has been isolated from mosquitoes, ticks, midges, swine and cattle in China, Vietnam, and Indonesia (Attoui et al., 2005; Nabeshima et al., 2008; Liu et al., 2010). *In China, BAV was isolated from various regions including Gansu, Shanxi, Inner Mongolia, Liaoning, Beijing, Hubei, and Yunnan* (Liu et al., 2010; Xia et al., 2018a; Xia et al., 2018b)

*Yunnan Province in the southwest of China, warm and rainy summers throughout most of the province are conducive to populations of biting flies such as mosquitoes and* midges. Therefore, Yunnan is one of the provinces with the most active insect-borne diseases in China, at least 13 types of mosquito-borne viruses and 4 types of *Culicoides*-borne viruses have been isolated from here (Xia et al., 2018b; Duan et al., 2022). In this study, 13 new BAV isolates were obtained from *Mosquitoes*, *Culicoides*, and *Ticks* collected from 7 counties in Yunnan Province between 2016 and 2023. The objective of this report is to present a genetic and phylogenetic analysis of the sequence data for Seg-1, -2, -5, -9 and -12 of the BAV strains, and to compare the levels of conservation and relationships between the complete coding sequences (CDSs) of them.

## 2 Materials and methods

### 2.1 Cell culture

C6/36 (*Aedes albopictus*) cells were used in this study and were propagated and maintained in 60% Dulbecco’s modified Eagle’s medium (HyClone) plus 30% Roswell Park Memorial Institute 1640 (HyClone), 5% fetal bovine serum (FBS, Invitrogen), 100 U/ml of penicillin, and 100 μg/ml of streptomycin, at 28°C in the presence of 5% CO_2_.

### 2.2 Sample collection and virus isolation

**Figure 1:**
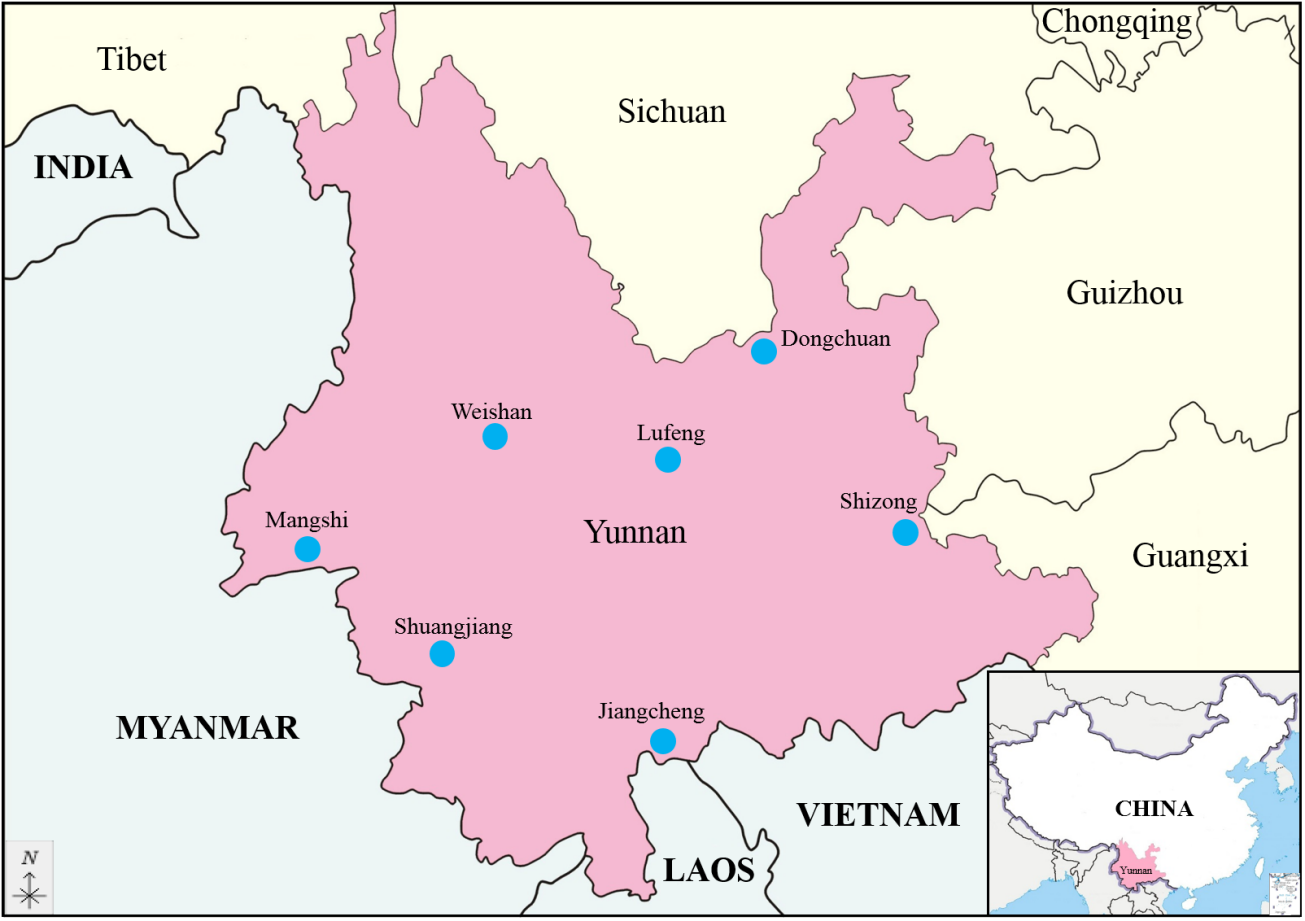
Geographic location of the seven sampling sites in Yunnan province, China (The map is drawn to a proportional scale of 1: 6 200 000).

Between 2016 and 2023, samples of mosquitoes and culicoides were collected from caprine and bovine shelters at night using light traps in the suburbs of Shuangjiang, Dongchuan, Shizong, Mangshi, Jiangcheng and Lufeng County in Yunnan Province. In addition, ticks were collected from on goats in Weishan County (**Figure 1**). Sample collection, classification and identification followed the guidelines described by Jinglin et al. (Wang et al., 2015). Virus was isolated from the homogenized liquid of the sample, as described by Jinglin et al. (Wang et al., 2017). Virus isolates were propagated in C6/36 cell cultures until approximately 90% of the monolayer showed complete cytopathic effects (CPE).

### 2.3 Viral dsRNA extraction and electropherotype analysis

Viral RNA was extracted from infectious C6/36 cells using RNAiso Plus (TaKaRa, Dalian, China) according to the manufacturer’s instructions. The separation of viral dsRNA from total RNA was conducted as described by Attoui et al. (Attoui et al., 2000b). As described previously, 6 μL viral RNA were taken for electropherotype analysis, and the remaining RNA were stored at −80°C for full-length amplification of cDNAs (Yang et al., 2023). Viral genomic segments were separated by 1% agarose gel electrophoresis (AGE) at 90 V for 4 h in 1× Tris-Acetate EDTA (TAE) buffer, stained with Goldview II (Solarbio, Beijing, China), and photographed by a Gel Doc™ XR+ System with Image Lab™ software (Bio-Rad, Hercules, CA, USA).

### 2.4 Full-length amplification of cDNAs and sequencing

BAV genomic segments were reverse-transcribed into cDNA using full-length amplification of cDNAs (FLAC) technique as described by Maan et al. (Maan et al., 2007). Briefly, the 3’ ends of each viral dsRNA were ligated to an ‘anchor-primer’, with a phosphorylated 5’ terminus, using T4 RNA ligase, followed by reverse transcription using a PrimeScriptTM II Reverse Transcriptase Kit (TaKaRa, Dalian, China). The resulting cDNAs were amplified using a primer (5-15-1) complementary to the ‘anchor-primer’ by high-fidelity PrimeSTAR® GXL DNA Polymerase (TaKaRa), the amplicons were analyzed by 1% AGE. The DNA products were sent to Magigen Company (Guangzhou, China) for Next-generation sequencing (NGS) using a HiSeq 2000 system, followed by reads preparation, reads/sequences checking, and contigs assembling for each genomic segment of the virus using the Soapnuke (v2.0.5), BWA (v0.7.17) and Megahit (v1.1.2) software, respectively (Li et al., 2016; Chen et al., 2018).

### 2.5 Sequence analysis and phylogenetic tree construction

Reference sequences of BAV strains and representative members of the *Seadornavirus* genus (Banna-like virus, Liao ning virus, Mangshi virus, and Kadipiro virus) were downloaded from GenBank on 16th February 2023 and are listed in **Table S1** (**Supplementary Material**). Open reading frames (ORFs) of BAV genomic segments were identified and translated into amino acid sequences using ORF-finder Home-NCBI (https://www.ncbi.nlm.nih.gov/orffinder/). Multiple alignments of consensus sequences were performed using CLUSTAL W program (Thompson et al., 1994). Nucleotide and amino acid (nt/aa) identities were calculated for each segment among the strains using MEGA (v6.06) and BioEdit Sequence Alignment Editor (v7.0.9.0). Phylogenetic trees were constructed in MEGA (v6.06) using CDSs with Neighbour-joining (NJ) methods in a pair-wise deletion, p-distance algorithm, and bootstrapped using 1000 replicates (Tamura et al., 2013).

## 3 Results

### 3.1 Virus isolation

**Figure 2:**
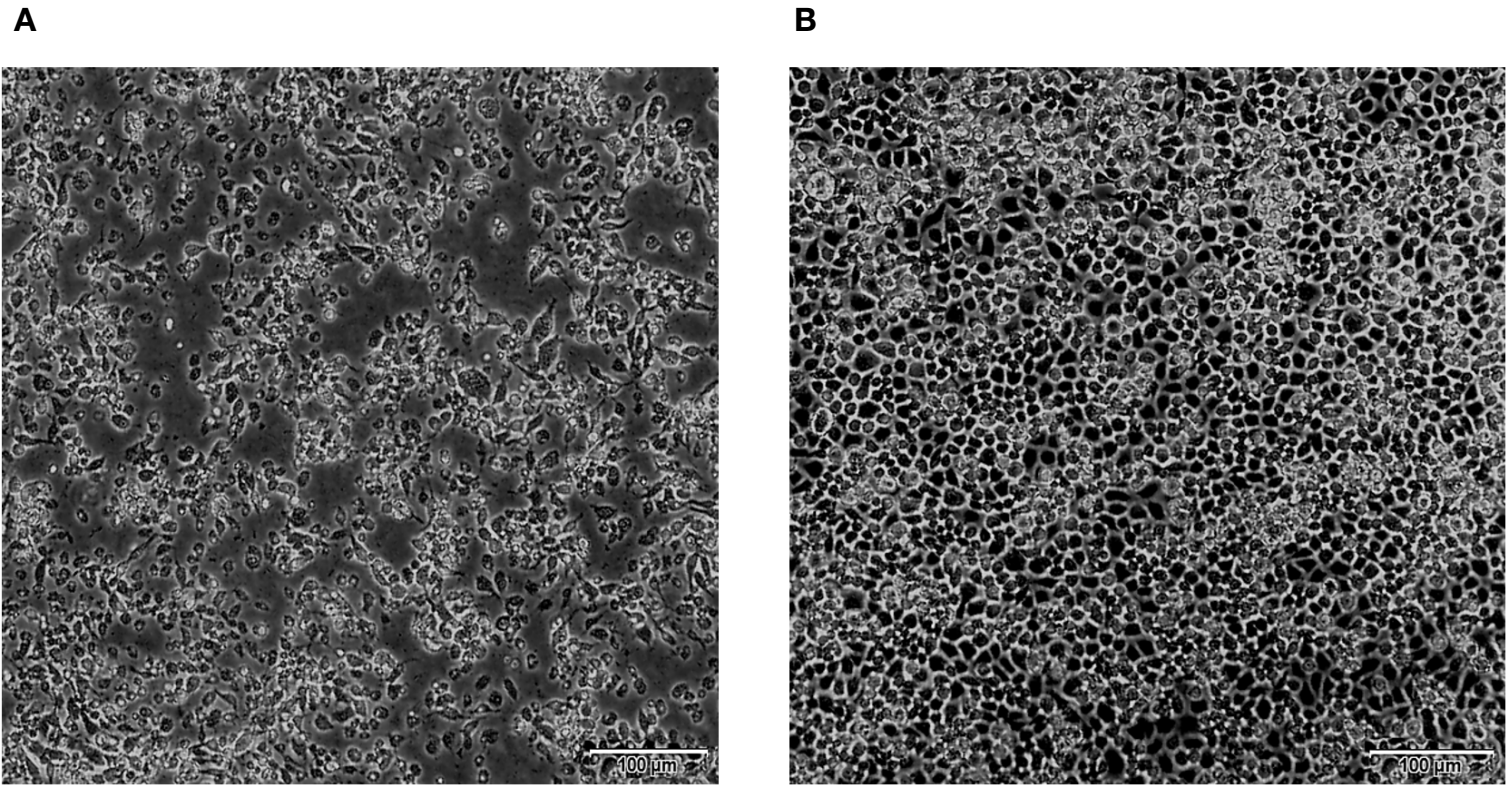
CPE caused by BAV in C6/36 cells at 72 h (×100). **(A)** 72 h post-infection, showing cell shrinking, aggregating, shedding, or cytolysis with eventual detachment from the growth surface. **(B)** Uninfected control cells (72 h).

A total of 11 594 culicoides collected from 3 locations were divided into 117 pools, 25 370 mosquitoes comprising 3 species collected from 5 locations were divided into 255 pools and 546 ticks collected from 1 location were divided into 22 pools, and were used for virus isolation (**Table S2**). A total of 13 BAV strains were obtained in Yunnan province during 2016-2023; 3 strains were isolated from culicoides, 9 strains from mosquitoes and 1 strain from ticks (**Table 1**). All strains can cause CPE in C6/36 cells at 72 h after inoculation and the characteristics of the CPE include cell shrinking, aggregating, shedding, or cytolysis with eventual detachment from the growth surface (**Figure 2**).

**Table 1:** Isolation details of thirteen BAV strains isolated from Yunnan Province, China.

Strain	Geographic origin (County)	Longitude and latitude	Altitude (m)	Host	Year collected	GenBank Accession no.
**JC_M4-2**	Jiangcheng	E101°52′29″, N 22°34′51″	1103.4	*Culex quinquefasciatus*	2016	OR476060 - OR476071
**MS_M166**	Mangshi	E98°29′27″, 24°20′37″	865.7	*Culex tritaeniorhynchus*	2017	OR476120 - OR476131
**MS_M140**	Mangshi	E98°29′27″, 24°20′37″	865.7	*Culex tritaeniorhynchus*	2017	OR476108 - OR476119
**MS_C38**	Mangshi	E98°29′27″, 24°20′37″	865.7	*Culicoides spp.*	2017	OR476096 - OR476107
**JC_M19-13**	Jiangcheng	E101°47′26″, N22°32′46″	831.6	*Culex tritaeniorhynchus*	2020	OR476084 - OR476095
**JC_C24-13**	Jiangcheng	E101°47′26″, N22°32′46″	831.6	*Culicoides spp.*	2020	OR476048 - OR476059
**DC_C18-1**	Dongchuan	E103°5′39″, N26°10′22″	1135.8	*Culex tritaeniorhynchus*	2020	OR476024 - OR476035
**DC_C55-33**	Dongchuan	E103°5′39″, N26°10′22″	1135.8	*Culex tritaeniorhynchus*	2020	OR476036 - OR476047
**SJ_M46**	Shuangjiang	E99°50′8″, N23°31′41″	1024.1	*Anopheles sinensis*	2022	OR476132 - OR476143
**SJ_M49**	Shuangjiang	E99°50′8″, N23°31′41″	1024.1	*Anopheles sinensis*	2022	OR476144 - OR476155
**LF_M39**	Lufeng	E102°5′23″, N25°5′39″	1650.8	*Anopheles sinensi*	2022	OR476072 - OR476083
**WS_T06**	Weishan	E100°20′13″, N25°14′57″	1883.3	*Tick*	2022	OR476168 - OR476179
**SZ_C36**	Shizong	E104°18′21″, N24°37′6″	942.6	*Culicoides spp.*	2023	OR476156 - OR476167

### 3.2 BAV genomic dsRNA electropherotype

**Figure 3:**
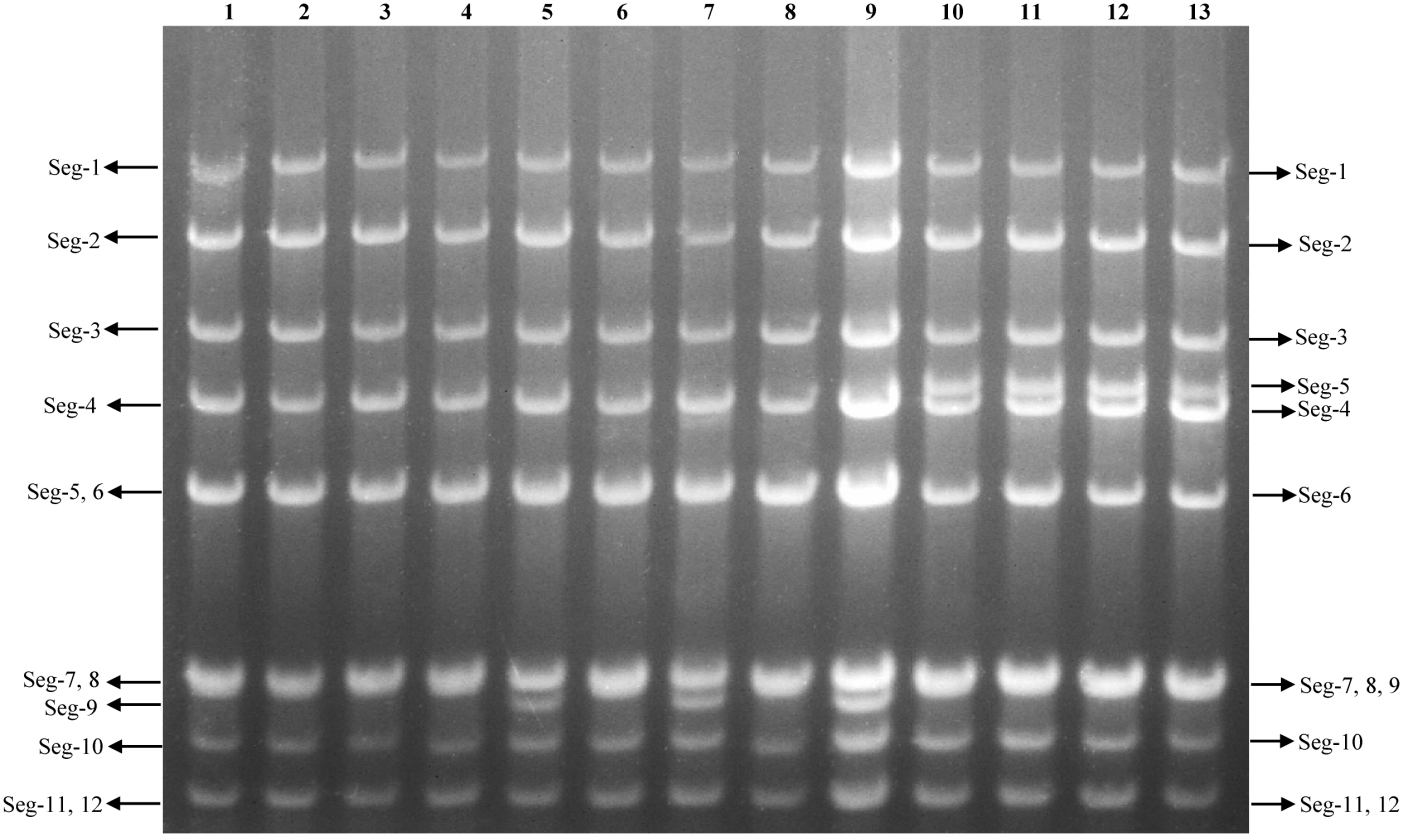
Agarose gel (1%) electrophoretic profile of the genomic dsRNAs of the 13 strains BAV. Lane 1=DC_C18-1; Lane 2=DC_C55-33; Lane 3=MS_C38; Lane 4=LF_M39; Lane 5=WS_T06; Lane 6=SZ_C36; Lane 7=MS_M166; Lane 8=JC_M4-2; Lane 9=MS_M140; Lane 10=SJ_M46; Lane 11=SJ_M49; Lane 12=JC_M19-13; Lane 13=JC_C24-13.

**Figure 4:**
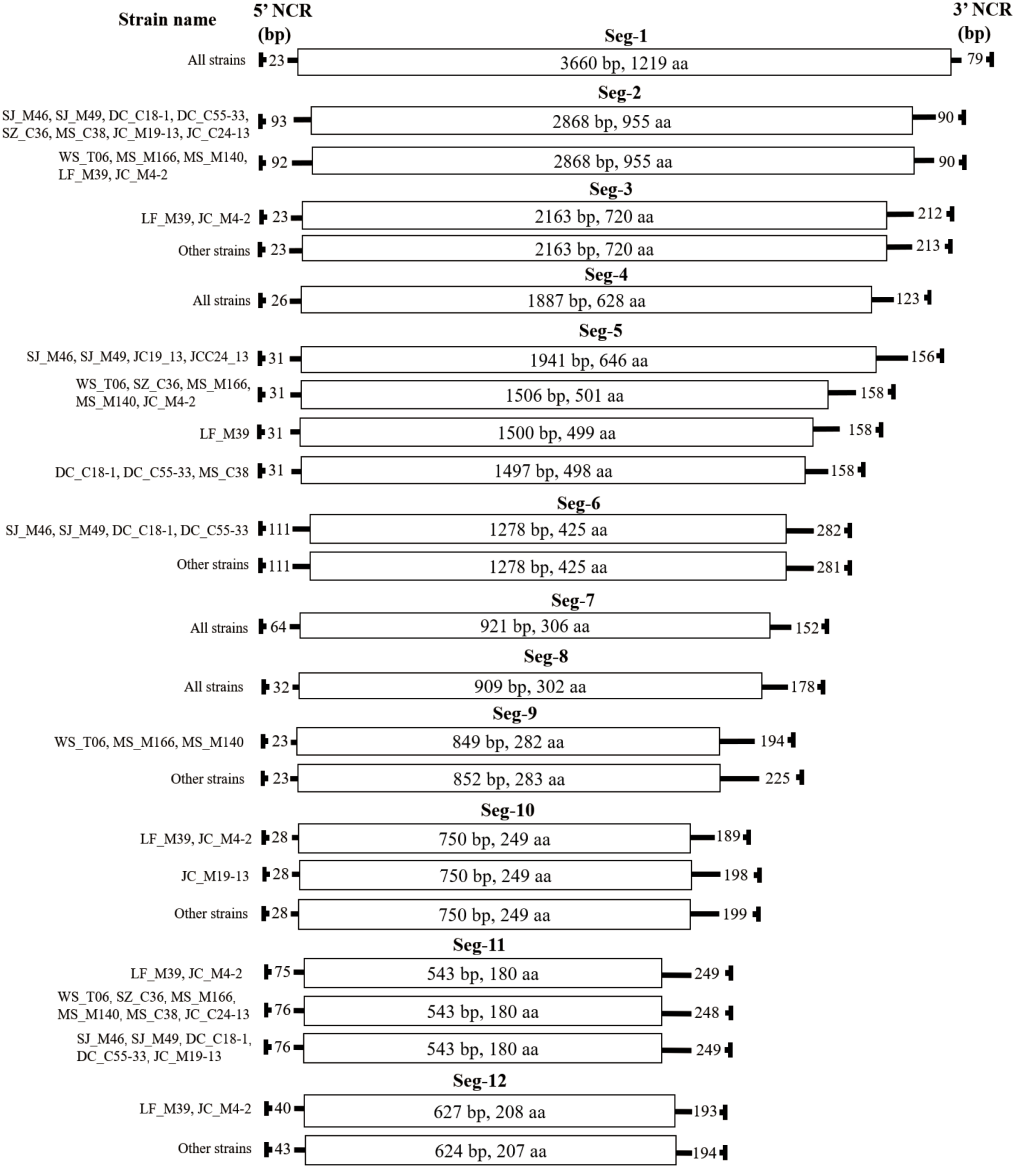
Schematic representation of the genome organisation of the 13 BAVs. The ORFs are shown as open boxes with segment names, and the nucleic acid and amino acid sizes of the ORF regions are annotated in the boxes. The 5 ‘and 3’ NCRs of each genomic segment are shown as solid lines, and the numbers on the solid lines indicate nucleotide length of the NCRs. The names of the strains are on the far left.

The AGE analysis shows that the majority of the BAVs genome segments migrate separately, with the exception of Seg-4/5, Seg-7/8/9, and Seg-11/12 which co-migrate in this gel system, respectively, generating a ‘6-6’ pattern (**Figure 3**). Sequencing and RNA electropherotypes have revealed an interesting anomaly in genome Seg-5 and Seg-9 of the BAVs. Seg-5 of four strains (SJ-M46, SJ-M49, JC19-13 and JCC24-13) were 2128 bp in length, a 435 bases nucleotide sequence (145 aa) was found to have inserted into their ORF at position 1311-1754 (**Table 2**). This represents a significant size difference in this RNA between these strains—a difference that is also clearly seen when the RNA electropherotypes are examined on 1% agarose (**Figure 4**). Moreover, in three strains (WS_T06, MS_M166 and MS_M140) Seg-7 and Seg-8 co-migrate, whilst Seg-9 runs separately. In other strains Seg-7,8 and 9 all co-migrate as one band (**Figure 3**). The sequencing results showed that the length of Seg-9 in the three strains was 1066 bp, with 34 bases nucleotides deletion at position 870-994, and the length of Seg-9 were 1100 bp in other strains (**Table 2**).

**Table 2:** Genetic analysis of virus genome segments and predicted proteins of the thirteen strains of BAV.

Segment	Segment length (bp)	Open reading frame (nt)	Size of protein (aa)	Protein molecular mass (kDa)	G+C content (%)	5’-teminal sequences	3’-teminal sequences	Stop-codon
**Seg-1**	3762	24-3683	1219	138.0–138.3	37.9–39.5	**GTAT**TA	ACT**GAC**	TAG
**Seg-2**	3050, 3051	93-2960, 94-2961	955	107.9–108.1	40.2–40.8	**GTAT**AA	C^C^/_T_C**GAC**	TAA
**Seg-3**	2398, 2399	24-2186	720	82.0–82.7	37.6–39.4	**GTAT**TA	ACT**GAC**	TAA
**Seg-4**	2036	27-1913	628	69.6–70.0	39.5–41.1	**GTAT**TT	ACC**GAC**	TAA
**Seg-5**	1686, 1689, 1695, 2128	32-1528, 32-1531, 32-1537, 32-1972	498, 499, 501, 646	55.1–71.8	39.0–40.9	**GTAT**T^T^/_A_	ACT**GAC**	TGA
**Seg-6**	1670, 1671	112-1389	425	48.4–48.6	41.0–42.5	**GTAT**TT	ACT**GAC**	TAA, TAG
**Seg-7**	1137	65-985	306	34.5–34.9	36.2–37.4	**GTAT**TT	ACT**GAC**	TAA, TAG
**Seg-8**	1119	33-941	302	32.8–33.0	32.8–33.0	**GTAT**TT	ACC**GAC**	TAG
**Seg-9**	1066, 1100	24-872, 24-875	282, 283	30.5–30.8	37.9–40.0	**GTAT**TT	ACC**GAC**	TAA
**Seg-10**	967, 976, 977	29-778	249	28.5–28.7	36.1–38.2	**GTAT**TT	ACT**GAC**	TAA, TAG
**Seg-11**	867, 868	76-618, 77-619	180	20.5–20.6	38.4–39.8	**GTAT**TA	ACT**GAC**	TAA
**Seg-12**	860, 861	41-667, 44-667	208, 207	23.9–24.1	37.6–38.8	**GTAT**TA	ACT**GAC**	TAA, TAG

Seg-2 length is 3050 bp in eight strains (SJ_M46, SJ_M49, DC_C18-1, DC_C55-33, SZ_C36, MS_C38, JC_M19-13 and JC_C24-13), and the length is 3051 bp in other five strains. Seg-3 length is 2399 bp in all strains, with the exception of two strains (LF_M39 and JC_M4-2), which are 2398 bp. Seg-5 length is 1686 bp in three strains (DC_C18-1, DC_C55-33 and MS_C38), the length is 1689 bp in only one strain LF_M39, the length is 1695 bp in five strains (WS_T06, SZ_C36, MS_M166, MS_M140 and JC_M4-2), and the length is 2128 bp in four strains (SJ_M46, SJ_M49, JC_M19-13 and JC_C24-13). Seg-6 length is 1670 bp in all strains, with the exception of four strains (SJ_M46, SJ_M49, DC_C18-1 and DC_C55-33), which are 1671 bp. Seg-9 length is 1100 bp in all strains, with the exception of three strains (WS_T06, MS_M166 and MS_M140), which are 1066 bp. Seg-10 length is 976 bp in only one strain JC_M19-13; the length is 967 bp in two strains (LF_M39 and JC_M4-2), and the length is 977 bp in other ten strains. Seg-11 length is 867 bp in eight strains (WS_T06, SZ_C36, MS_M166, MS_M140, MS_C38, JC_C24-13, LF_M39, JC_M4-2), the length is 868 bp in other five strains. Seg-12 length is 861 bp in all strains, with the exception of two strains (LF_M39 and JC_M4-2), which are 860 bp. More details can be found in the **Table S3**.

Conserved nucleotide sequences in 5′- and 3′-terminals are shown in bold.

### 3.3 Full genome sequences

​Next-generation sequencing was performed on an Illumina Hiseq platform using a paired-end method. ​Raw reads ranging from 116 512 618 to 116 721 464 were obtained for each of the 13 BAVs. After filtration of low-quality sequences and adapter sequences, *de novo* assembly were conducted to construct a consensus sequence for individual genome segments of each strain. Nearly 99.48% to 99.96% of qualified reads for each strain were assembled into 12 contigs, corresponding to 12 genomic segments, respectively. All of the sequences discussed refer to the positive strand, are written in a 5′–3′ orientate on and are submitted to the NCBI GenBank. Sequence data and GenBank accession number from each virus are provided in **Table S3**.

### 3.4 Genome organization and characteristics of the thirteen strains of BAV

The characteristics of genome Seg-1 to Seg-12 of the 13 BAV strains are shown in **Table 2** and **Figure 4**. The size of genome Seg-1, -4, -7 and -8 is highly conserved for all of the viruses studied, and are 3762, 2036, 1137 and 1119 bp, respectively. The rest of the genome segments are not conserved in length. Among the rest of the non-highly conserved segments, the coding sequence length and protein size varied only in Seg-5, -9 and -12. Analysis of the 5’ and 3’ noncoding regions (NCRs) showed that the BAV shares a highly conserved terminal nucleotide sequence at 5’- and 3’- NCRs (5’-GTAT and 3’-GAC, respectively) in each of the 12 genome segments. In the 13 BAVs genome segments, the 5’ NCRs are shorter than the 3’ NCRs, only Seg-2 has a longer 5’ NCR (92 or 93 bp) than 3’ NCR (90 bp). The 5’ and 3’ NCRs of the them comprised 12.84% to 13.13% of the total genome, respectively, and the G + C content was 39.23% to 39.6% (Data not displayed). Stop-codon usage was not conserved within Seg-6, -7, -10 and -12 and included TAA, TAG.

### 3.5 Genetic and phylogenetic characteristics of Seg-1/VP1

**Figure 5:**
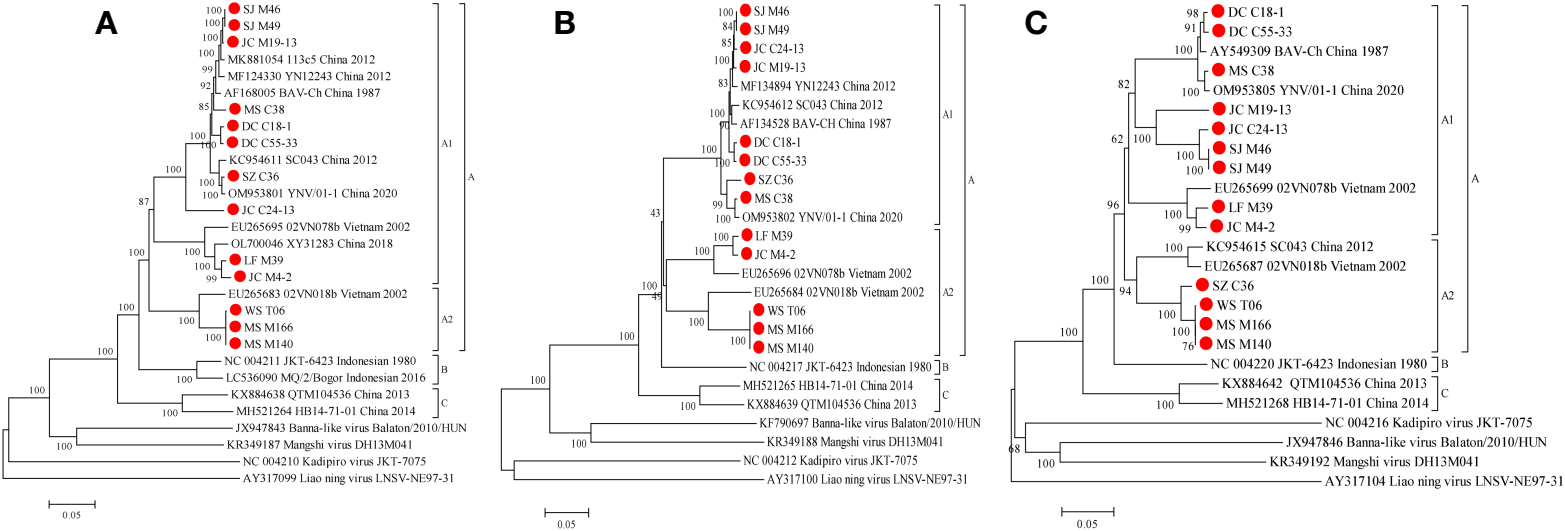
Phylogenetic analysis based on the CDSs of VP1 **(A)**, VP2 **(B)** and VP5 **(C)** of the 13 BAV strains and other members of the genus *Seadornavirus*. Neighbour-joining tree was constructed using p-distance determination algorithm in MEGA 6 with 1,000 bootstrap replicates. Complete coding genome of each reference BAV strains was represented as ‘GenBank accession number_ Strains number_ Country (region)_ year of isolation’. Outgroup virus were represented as ‘GenBank accession number_ Virus name_ Strains number’. The isolates in this study are depicted by red dots.

We conducted a phylogenetic analysis of the VP1 CDSs of the 13 BAVs and other strains isolated elsewhere. The VP1 nucleotide (nt) and amino acid (aa) sequence identity is ≥75.3% (μ=86.2%) and ≥87.5% (μ=94.9%), respectively (**Table 3**). BAVs can be genotypically classified into three groups (A, B and C) based on VP1 CDSs and the 13 strains were sorted into Groups A (**Figure 5A**). In Group A, percentage nt identity is ≥81.9% (μ=89.3%) and aa identity is ≥93.6% (μ=97.6%). In Group A1, ten BAV strains in this study clustered together with the BAV strains from Yunnan in China and Vietnam (02VN078b) to formed a large branch, and shared 83.7%-99.4%/94.5%-99.9% nt/aa sequence identity with them (**Table S4**). In Group A2, three strains (WS_T06, MS_M166 and MS_M140) were closely related to 02VN018b strain isolated from Vietnam and showed 94.2%-94.3% nt and 98.6%-98.7% aa identities, respectively (**Table 3**).

**Table 3:** Summary of percentage sequence identities of nucleotide (nt) and amino acid (aa) for VP1, VP2, VP5, VP9 and VP12 for Group A, Group B, Group C and all strains respectively.

	Group A1		Group A2		Group A		Group B		Group C		All strains	
	NT	AA	NT	AA	NT	AA	NT	AA	NT	AA	NT	AA
**Seg-1**	≥83.7	≥94.5	≥94.2	≥98.6	≥81.9	≥93.6	94.1	97.2	89.4	95.3	≥75.3	≥87.0
	*μ*=91.9	*μ*=97.4	*μ*=97.1	*μ*=99.3	*μ*=89.3	*μ*=97.6	*μ*=94.1	*μ*=97.2	*μ*=89.4	*μ*=95.3	*μ*=86.2	*μ*=94.9
**Seg-2**	≥95.4	≥98.3	≥82.1	≥90.6	≥80.7	≥90.5	-	-	94.0	98.2	≥74.5	≥85.9
	*μ*=97.5	*μ*=99.2	*μ*=87.8	*μ*=94.2	*μ*=88.6	*μ*=95.1	-	-	*μ*=94.0	*μ*=98.2	*μ=*85.8	*μ=*93.5
**Seg-5**	≥62.8	≥67.5	≥87.0	≥93.6	≥62.3	≥66.7	-	-	91.2	94.8	≥53.5	≥58.0
	*μ*=76.80	*μ*=81.2	*μ*=92.5	*μ*=96.3	*μ*=77.5	*μ*=82.3	-	-	*μ*=91.2	*μ*=94.8	*μ*=75.2	*μ*=80.3
**Seg-9**	≥79.8	≥84.4	≥93.7	≥95.4	≥79.8	≥84.4	≥82.3	≥88.6	99.6	99.6	≥48.5	≥38.2
	*μ*=86.9	*μ*=91.0	*μ*=96.5	*μ*=97.8	*μ*=87.4	*μ*=90.8	*μ*=88.1	*μ*=92.9	*μ*=99.6	*μ*=99.6	*μ*=78.9	*μ*=80.4
**Seg-12**	≥92.9	≥92.7	≥89.9	≥91.3	≥87.0	≥86.9	≥95.5	≥96.6	≥93.5	≥95.6	≥76.4	≥79.7
	*μ*=95.8	*μ*=95.5	*μ*=95.60	*μ*=96.7	*μ*=93.5	*μ*=94.9	*μ*=97.5	*μ*=98.3	*μ*=93.5	*μ*=95.6	*μ*=91.2	*μ*=92.6

*μ* is the mean, - is not available.

### 3.6 Genetic and phylogenetic characteristics of Seg-2/VP2

Phylogenetic analyses of VP2 CDSs demonstrate that the nt and aa sequence identity is ≥74.5% (μ=85.8%) and ≥85.9% (μ=93.5%), respectively (**Table 3**). BAVs can be genotypically classified into three groups (A, B and C), and the 13 strains were sorted into Groups A (**Figure 5B**). In Group A, percentage nt identity is ≥80.7% (μ=88.6%) and aa identity is ≥90.5% (μ=95.1%), respectively (**Table 3**). In Group A1, eight strains of BAV in this study clustered together with the BAV strains isolated from Yunnan, China to formed an independent branch, and shared 95.4%-99.3%/98.3%-99.8% nt/aa sequence identity with them (**Table S5**). In Group A2, two strains (LF_M39 and JC_M4-2) were closely related to 02VN078b strain (94.2%-98.8% nt and 98.8%-99.7% aa identities, respectively), three strains (WS_T06, MS_M166 and MS_M140) were closely related to 02VN018b strain (90.2%-100% nt and 96.8%-100% aa identities, respectively), and eventually formed two separate branches within the Group A2 (**Table S5**). The two strains (02VN078b and 02VN018b) were isolated from Vietnam.

### 3.7 Genetic and phylogenetic characteristics of Seg-5/VP5

Phylogenetic analyses demonstrate that the VP5 nt and aa sequence identity is ≥53.5% (μ=75.2%) and ≥58.0% (μ=80.3%), respectively (**Table 3**). BAVs can be genotypically classified into three groups (A, B and C), and the 13 strains were sorted into Group A (**Figure 5C**). In Group A1, percentage nt identity is ≥62.8% (μ=76.8%) and aa identity is ≥67.5% (μ=81.2%), respectively (**Table 3**). Furthermore, two strains (LF_M39 and JC_M4-2) were closely related to 02VN078b strain isolated from Vietnam (94.8%-96.8% nt and 96.2%-97.4% aa identities, respectively), three strains (DC_C18-1, DC_C55-33 and MS_C38) were closely related to two strains (BAV-Ch and YNV/01-1) isolated from China (97.7%-99.2% nt and 98.3%-99.5% aa identities, respectively), and four strains (JC_M19-13, JC_C24-13, SJ_M46 and SJ_M49) clustered into a subgroup (88.5%-100% nt and 93.3%-100% aa identities, respectively, **Table S6**). In Group A2, four strains (SZ_C36, WS_T06, MS_M166 and MS_M140) were closely related to two strains (SC043 and 02VN018b) but formed a separated branch, and shared 87.0%-87.6%/93.6%-94.6% nt/aa sequence identity with them (**Table S6**).

### 3.8 Genetic and phylogenetic characteristics of Seg-9/VP9

**Figure 6:**
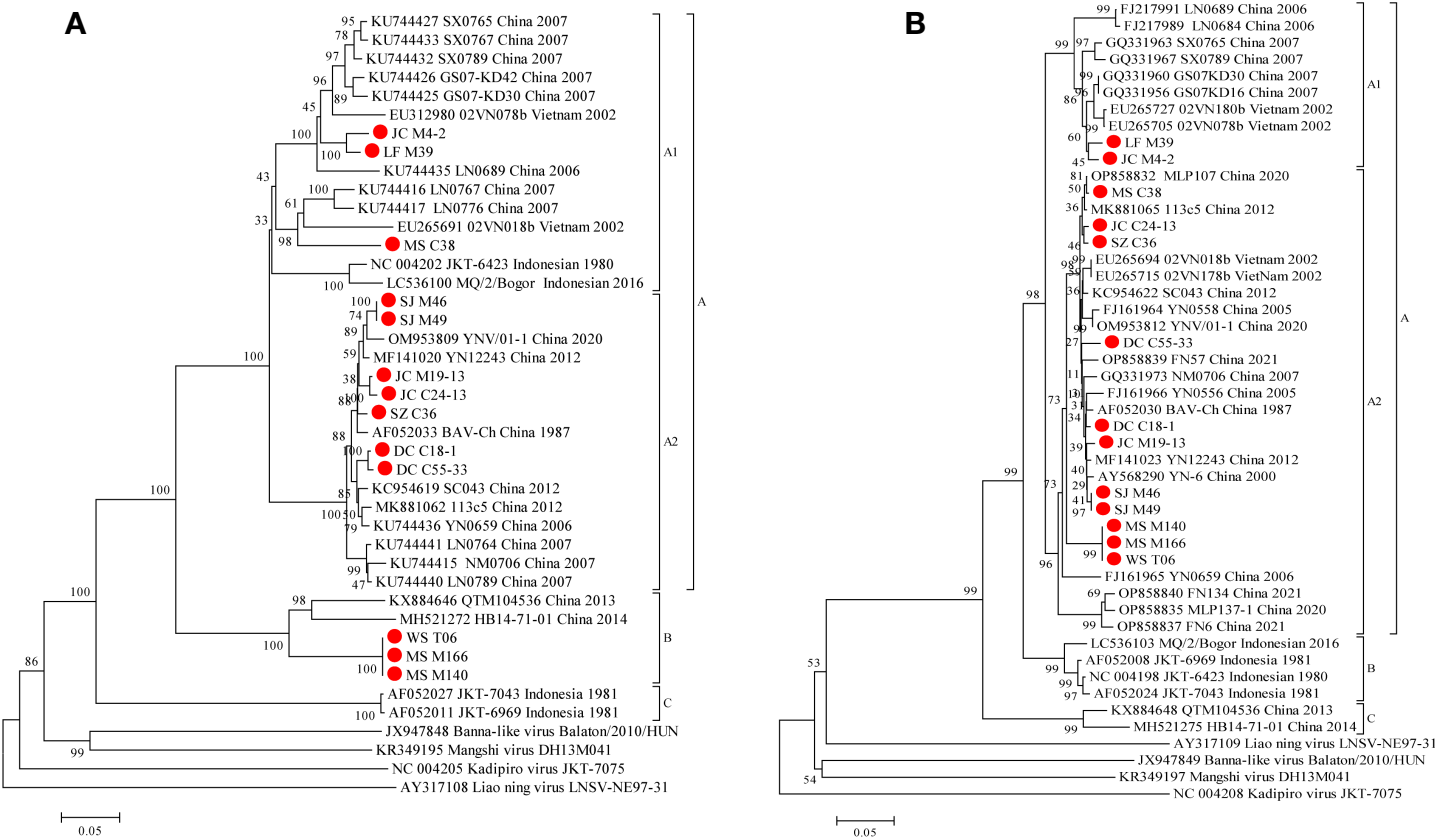
Phylogenetic analysis based on the CDSs of VP9 **(A)** and VP12 **(B)** of the 13 BAV strains and other members of the genus *Seadornavirus*. Neighbour-joining tree was constructed using p-distance determination algorithm in MEGA 6 with 1,000 bootstrap replicates. Complete coding genome of each reference BAV strains was represented as ‘GenBank accession number_ Strains number_ Country (region)_ year of isolation’. Outgroup virus were represented as ‘GenBank accession number_ Virus name_ Strains number’. The isolates in this study are depicted by red dots.

Phylogenetic analyses demonstrate that the VP9 nt and aa sequence identity is ≥48.5% (μ=78.9%) and ≥38.2% (μ=80.4%), respectively (**Table 3**). BAVs can be genotypically classified into three groups (A, B and C), and the 13 strains were sorted into Groups A and B, respectively (**Figure 6A**). In Group A, percentage nt identity is ≥79.8% (μ=87.4%) and aa identity is ≥84.4% (μ=90.8%), respectively (**Table 3**). In Group A1, three strains (LF_M39, JC_M4-2, and MS_C38) were clustered together with strains from Gansu, Shanxi, and Liaoning in China and two strains (02VN078b and 02VN018b) from Vietnam to formed a separated branch, and shared 81.3%-92.2%/85.1%-98.5% nt/aa identity with them. In Group A2, seven strains in this study clustered together with BAV strains isolated from Yunnan in China formed a separate branch, and shared 95.5%-98.1%/96.8%-99.6% nt/aa identity with them. In Group B, three strains (WS_T06, MS_M166, and MS_M140) were closely related to two strains (QTM104536 and HB14-71-01) from Hubei in China and shared 82.3%-82.8%/88.6%-90.7% nt/aa sequence identity with them (**Table S7**).

### 3.9 Genetic and phylogenetic characteristics of Seg-12/VP12

Phylogenetic analyses demonstrate that the VP12 nt and aa sequence identity is ≥76.4% (μ=91.2%) and ≥79.7% (μ=92.6%), respectively (**Table 3**). BAVs can be genotypically classified into three groups (A, B and C), and the 13 strains were sorted into Groups A (**Figure 6B**). In Group A, percentage nt identity is ≥87.0% (μ=93.5%) and aa identity is ≥86.9% (μ=94.9%), respectively (**Table 3**). In Group A1, two strains (LF_M39 and JC_M4-2) clustered together with BAVs isolated from Gansu, Shanxi, and Liaoning in China and two strains (02VN180b and 02VN078b) isolated from Vietnam, and shared 92.9%-96.8%/92.7%-97.1% nt/aa sequence identity with them (**Table S8**). In Group A2, except for a strain (NM0706) isolated from Inner Mongolia in China, and two strains (02VN178b and 02VN018b) isolated from Vietnam, all other strains were isolated from Yunnan in China, and showed ≥89.9% (μ=95.6%) nt and ≥91.3% (μ=96.7%) aa sequence identities, respectively (**Table 3**). Meanwhile, eleven strains that isolated from different blood-sucking insects (*Culicoides, Mosquitoes and Ticks*) in this study shared 90.2%-99.8%/92.7%-100% nt/aa sequence identity with other strains in Group A2 (**Table S8**).

### 3.10 Phylogenetic analysis of other structural and non-structural proteins of the 13 BAVs

Phylogenetic trees constructed for the CDSs of other structural and non-structural proteins of the 13 BAVs (**Figure S1**). The results show that the VP3, VP4, VP6 and VP10 all show similar relationships to those seen in VP1, VP2, VP5 and VP12 proteins, with distinct monophyletic groups for genotypes, the Chinese and Vietnamese strains clustered in Group A, the Indonesian strains in Group B, and the isolates from Hubei, China in Group C. Although BAVs can be genotypically classified into three groups (A, B and C) based on VP8 and VP11 CDSs, isolates from China, Vietnam and Indonesia are included in Group A, while LF_M39, JC_M4-2, and the Vietnamese strain (02VN078b) clustered in Group B on the VP8 phylogenetic tree, two Indonesian strains (JKT-6423 and MQ/2/Bogor) clustered in Group B on the VP11 phylogenetic tree. However, the phylogenetic tree of VP7 CDSs can be divided into four groups (A, B, C and D), with two Indonesian strains clustered in groups B and D, respectively.

## 4 Discussion

In this study, we conducted a phylogenetic analysis of the CDSs of the VP1, VP2, VP5, VP9 and VP12 of the 13 BAVs and other strains isolated elsewhere. There was no significant difference in the sequence identities of nucleic acids and amino acids between BAVs isolated from different blood-sucking insects. E. g. the BAVs isolated from different species of mosquitoes can apparently be clustered together. The WS_T06 isolated from ticks has the same nucleotide and amino acid sequence lengths as the BAVs (MS_M166 and MS_M166) isolated from mosquitoes, and closer nucleotide and amino acid sequence identities. The same was true for JC_C24-13 isolated from culicoides and SJ_M46 isolated from mosquitoes. This result of this study is the same as that reported by Liu Hong et al. and Song et al. (Liu et al., 2016a; Song et al., 2017), there are no obvious species barriers exist in the BAVs population. In addition, BAV strains isolated from various blood-sucking insects clustered significantly according to their geographical distribution. Geographical separation may enable the BAVs in different region to acquire unique mutations, some of which might make them particularly well suited to transmission and survival in their respective local ecosystem, which has over time led to the evolution of distinct geographical strains or genotypes.

Seg-1 and Seg-2, which encode the inner core proteins VP1(Pol) and VP2 (T2) of BAV particles, respectively, are a highly conserved and an important marker for species identification across the family *Reoviridae* (Jaafar et al., 2005b; Belaganahalli et al., 2015). The phylogenetic analysis of VP1 and VP2 showed that BAVs can be genotypically classified into three groups: Group A, B and C, and all the 13 strains in the study were sorted into Group A. In Group A, nucleotide sequence identity in VP1 and VP2 are ≥81.9% (μ=89.3%) and ≥80.7% (μ=88.6%), respectively, which increases to ≥93.6% (μ=97.6%) and ≥90.5% (μ=95.1%) at the amino acid level, reflecting the presence of synonymous mutations and the presence of selective pressure to maintain protein integrity. However, these strains in Group A isolated from a limited geographic region (Yunnan in China and Vietnam), and do not therefore represent the global conservation acting on these proteins. When strains from Hubei in China and Indonesia are considered together, the overall level of aa identity in VP1 and VP2 drop to ≥87.0% (μ=94.9%), and ≥85.9% (μ=93.5%), respectively.

Seg-5, which encodes the non-structural protein VP5, shows a significant size and identity difference in coding sequence. The overall level of nt/aa identity in VP5 were ≥53.5% (μ=75.2%) and ≥58.0% (μ=80.3%), respectively, suggesting that there are fewer essential domains required to preserve protein function. However, phylogenetic tree of VP5 show similar relationships to those seen in VP1 and VP2 proteins, with distinct monophyletic groups, and all the 13 strains were sorted into Group A. Seg-12 encodes the dsRNA binding protein (VP12), which is a non-structural protein (Jaafar et al., 2005b; Liu et al., 2016a). In this study, the phylogenetic tree based on the CDSs of Seg-12 is similar to that previously reported (Liu et al., 2016b; Song et al., 2017; Xia et al., 2018a; Li et al., 2022), that the BAV strains can be divided into three genotypes: A, B and C. Isolates from China and Vietnam are included in Group A, Group B comprises Indonesian strains, and Group C consists of two strains obtained from central China, Hubei province. Additionally, Group A can be divided into two subgroups; the Group A1 strains were isolated from north China, and the Group A2 strains were isolated from south China and Vietnam. However, two strains (LF_M39 and JC_M4-2), like two Vietnam strains (02VN180b and 02VN078b), are clustered in subgroup A1 along with the strains isolated from northern China. These observations suggest that the distribution of BAV in Yunnan, China is wider than previously recognized and may be increasing.

The structural protein VP9 that encoded by Seg-9, is an outer-coat attachment protein and involved in virus attachment to the host-cell surface and subsequent internalization (Jaafar et al., 2004; Jaafar et al., 2005a). Seg-9/VP9 is the most variable of the BAV genome segments with ≥48.5% (μ=78.9%) identity at the nucleotide level, and only ≥38.2% (μ=80.4%) at the amino acid level, reflecting the presence of non-synonymous mutations in the coding sequence of VP9, and of the presence of selective pressure which presumably favours genetic variants. It was previously reported that BAV can be divided into three genotypes based on phylogenetic analysis VP9 or VP12 coding sequence (Liu et al., 2016b; Xia et al., 2018a; Li et al., 2022). In this study, the phylogenetic relationships seen for both VP9 and VP12 are similar (**Figure 4**), but there are some significant differences. In VP9 phylogenetic tree, some strains did not cluster together according to geographical distribution as seen in VP12 tree, such as two Indonesian strains (JKT-6423 and MQ/2/Bogor) clustered in subgroup A1, and three Yunnan strains (WS_T06, MS_M166 and MS_M140) and Hubei strains clustered in Group B, suggesting that there are some different forces of selection acting to shape the relationships seen in the both proteins.

The location and role of VP9 mean that it is exposed to the host’s immune system more than any other protein, which places the gene coding sequence (Seg-9) for this protein under selective pressure to adapt in order to evade neutralising antibodies. VP12 is a non-structural protein make it less of a target for neutralising antibodies and may place it under relatively less selective pressure to change than VP9. Jaafar et al. have previously reported that native and recombinant VP9 proteins of BAV-Ch (genotype A) failed to cross-react with anti-VP9 of BAV-In6969 (genotype B) and vice versa, and indicated that VP9 is both antigenically variable and can be used to identify two serotypes, A and B (Jaafar et al., 2004; Jaafar et al., 2005a). Taken together, we cautiously conclude that the phylogenetic relationships of the VP9 CDSs may be influenced by the selective pressures from host’s neutralising antibodies. However, further study is needed to prove this conclusion.

The genome-segments of the same orbivirus, usually show a high level of conservation in their sizes or molecular weights, and have a consistent electropherotype when analyzed by 1% AGE (Eaton and Gould, 1987). The dsRNA profiles of the 13 BAVs in 1% AGE exhibit three distinct electropherotype, with large differences in the sizes and migration pattern of Seg-5 and Seg-9, is a novel observation and to our current knowledge is unique to BAV within the genus *Seadornavirus* (**Figure 2**). Song et al. (2017) previously noted a difference in the genome migration pattern of two BAV strains which isolated from *Culicoides* and *Mosquitoes*, respectively (Song et al., 2017). This suggests that the genome migration pattern of BAV is not as conserved as that of orbivirus. ​It may be that, as has been previously reported, BAV is an emerging virus at a stage that involves rapid evolution (Liu et al., 1016). Four strains (SJ-M46, SJ-M49, JC-M19-13, and JC-C24-13) with the identical changes in the length of Seg-5 isolated from Shuangjiang and Jiangcheng County along the Sino-Burmese and Sino-Laotian borders (**Figure 1**), respectively. Moreover, three strains (WS_T06, MS_M166 and MS_M140) with the same changes in the length of Seg-9 isolated from Weishan and Mangshi County (which are geographically close), respectively. This suggests that these changes in the genome size of BAVs may be related to geographical location. But it is not even clear what selective forces, if any, are at work to shape these mutations.

BAV is thought to be a mosquito-borne virus that may be transferred by wind among infected mosquitoes (Liu et al., 2010). Thus far, BAV have been isolated from 10 mosquito species belonging to 3 genera (*Aedes*, *Anopheles*, and *Culex*) collected in Indonesia, Vietnam, South Korea, and China (Gansu, Shanxi, Inner Mongolia, Liaoning, Beijing, Hubei and Yunnan) (Nabeshima et al., 2008; Xia et al., 2018a; Supriyono et al., 2020). In this study, 9 strains of BAV strains were isolated from two genera of mosquitoes (*Anopheles* and *Culex*). Therefore, enhanced monitoring and long-term surveillance of BAV carried by mosquitoes is important for understanding the prevalence and distribution of BAV. Song et al. previously reported the isolation of a BAV strain (YN12243) from *Culicoides* collected in 2012 in the China and Myanmar border area of Yunnan (Song et al., 2017). Additionally, Li et al. isolated a strain (SC043) from *Culicoides* collected from Shizong county of Yunnan in 2012 (Li et al., 2021). Duan et al. then isolated a strain (YNV/01-1) from *Culicoides* without blood meals collected from the same area in 2020, which is the second time that BAV has been isolated in this region (Duan et al., 2022). In this study, three BAV strain were isolated from *Culicoides* collected in Mangshi, Jiangcheng and Shizong counties of Yunnan. So far, there have been no reports of isolated BAV from *Culicoides* collected elsewhere than Yunnan. However, members of the genus *Culicoides* are widely distributed in China, and have been implicated as potentially important vectors for many arboviruses (Mellor et al., 2000). ​Therefore, it is necessary to strengthen surveillance of *Culicoides* carrying BAV and further investigate whether *Culicoides* are natural vectors for BAV.

Li et al. previously reported the isolation of seventeen BAV strains from ticks in the northern and western regions of Xinjiang, China, in 1990, but did not describe whether these ticks had sucked the blood or not. (published in Chinese; sequence information not available in GenBank) (Li et al., 1992). In 2022, we once again isolated a BAV strain from ticks collected on a goat in Weishan county. ​However, during sample processing, it was found that the ticks had ingested goat blood, so it is uncertain whether the virus was isolated from the ticks themselves or the goat blood. Further study is required to determine whether the ticks are competent vectors for BAV.

## 5 Conclusions

​In the study, a comprehensive sequence dataset was made available for BAV and demonstrated the widespread prevalence of multiple strains in Yunnan Province. We have also provided genetics and phylogenetics details on VP1, VP2, VP5, VP9 and VP12 genes. Interestingly, BAV strains isolated from different vectors phylogenetically clustered together according to geographical distribution than with their isolated host-species. Furthermore, the phylogenetic relationship of VP9 is somewhat different from that of VP12, which may be caused by more selective pressure on VP9. The data obtained herein would be beneficial for the surveillance of evolutionary characteristics of BAV in China and neighboring countries as well as extend the knowledge about its genomic diversity and geographic distribution.

## Data availability statement

The datasets presented in this study can be found in online repositories. The names of the repository/repositories and accession number(s) can be found in the article/**Supplementary Material**.

## Ethics statement

The manuscript presents research on animals that do not require ethical approval for their study.

## Author contributions

ZY: Data curation, Formal Analysis, Writing – original draft. YH: Data curation, Formal Analysis, Writing – original draft. YC: Data curation, Formal Analysis. MJ: Data curation, Formal Analysis. NL: Data curation, Formal Analysis. SL: Data curation, Formal Analysis. JW: Funding acquisition, Methodology, Supervision, Writing – review & editing.
