# Supplementary material for: Full genome characterization and evolutionary analysis of Banna virus isolated from Culicoides, mosquitoes and ticks in Yunnan, China
Source: Front Cell Infect Microbiol. 2023 Nov 16;13:1283580. doi: 10.3389/fcimb.2023.1283580 (PMC10687475; doi:10.3389/fcimb.2023.1283580)
Supplement: Supplementary file 1 [file DataSheet_1.zip › Supplementary Material (20231016)/Figure S1. Phylogenetic analysis based on the CDSs of VP3 (a), VP4 (b), VP6 (c), VP7 (d), VP8 (e), VP10 (f) and VP11 (g) of the 13 BAV strains and other members of the genus Seadornavirus.pdf]

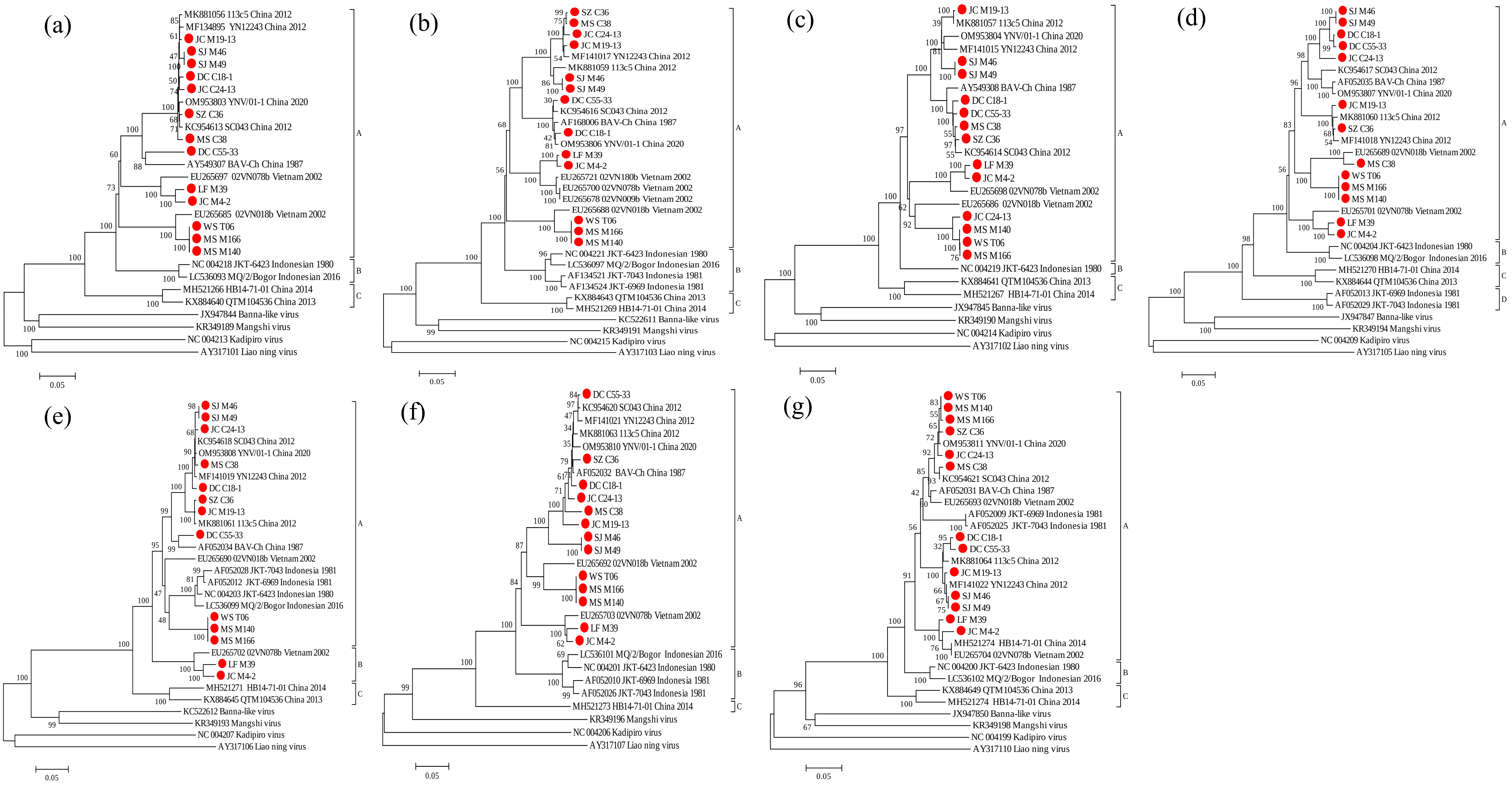

FIGURE S1

Phylogenetic analysis based on the CDSs of VP3 (a), VP4 (b), VP6 (c), VP7 (d), VP8 (e), VP10 (f) and VP11 (g) of the 13 BAV strains and other members of the genus *Seadornavirus*. Neighbour-joining tree was constructed using p-distance determination algorithm in MEGA 6 with 1,000 bootstrap replicates. Complete coding genome of each reference BAV strains was represented as 'GenBank accession number_ Strains number_ Country (region)_ year of isolation'. Outgroup virus were represented as 'GenBank accession number_ Virus name'. The isolates in this study are depicted by red dots.
